# Chemistry for electron-induced nanofabrication

**DOI:** 10.3762/bjnano.9.124

**Published:** 2018-04-30

**Authors:** Petra Swiderek, Hubertus Marbach, Cornelis W Hagen

**Affiliations:** 1 University of Bremen, Faculty 2 (Chemistry/Biology), Institute of Applied and Physical Chemistry, Leobener Straße 5, 28334 Bremen, Germany; 2 Lehrstuhl für Physikalische Chemie II and Interdisciplinary Center for Molecular Materials (ICMM), Friedrich-Alexander-Universität Erlangen-Nürnberg, Egerlandstr. 3, 91058 Erlangen, Germany,; 3 Department of Imaging Physics, Delft University of Technology, Lorentzweg 1, 2628CJ Delft, The Netherlands

**Keywords:** electron-induced chemistry, FEBID, FEBIP, nanofabrication, nanolithography

Electron-induced chemistry of volatile precursor molecules is central to a novel class of gas-assisted nanolithographic techniques, subsumed as focused electron beam induced processing (FEBIP) [1–2]. FEBIP has emerged with the availability of extremely narrow focused electron beams in electron microscopy. These beams can be used to induce, on a very small area, chemical reactions of adsorbed precursor molecules that either lead to etching of the underlying surface or deposition of material. The latter additive variant of FEBIP is focused electron beam induced deposition (FEBID), a powerful direct-write technique for nanofabrication capable of producing structures that range from 0D dots with a diameter of less than 1 nm [3] to arbitrarily shaped free-standing 3D structures with sub-10 nm size [1–2] or fascinating complexity [4–5]. These structures can serve as devices that give access to a wealth of new physical quantum-scale phenomena and thus novel functionalities [2]. However, their performance depends decisively on the precise control of the electron-induced precursor chemistry that is fundamental to FEBID.

In FEBID, the precursor molecules are dosed into an electron microscope where they adsorb on a surface and are decomposed under the tightly focused electron beam to yield a solid deposit. The precursor consists of elements that are desired in the deposit and of ligands which provide the molecules with sufficient volatility to be handled via a gas injection system. Ideally, the precursor molecule dissociates completely under the impact of an impinging electron so that the desired nonvolatile elements remain on the surface while the ligands desorb and are pumped out of the vacuum chamber. However, until recently, FEBID has relied nearly exclusively on precursors that were developed specifically for chemical vapor deposition (CVD), which is a thermally driven process [6]. Consequently, these precursors are optimized with respect to thermal chemistry and do not necessarily perform well in the electron-driven FEBID process. In fact, they often experience incomplete fragmentation so that material from the ligands is co-deposited along with the desired elements. This unintended contamination often deteriorates the targeted properties of the deposit and thus impedes the progress of FEBID technology [7–8]. Novel molecular precursors and improved processes are thus needed to advance FEBID to its full potential.

In fact, the composition and spatial resolution of deposits fabricated by FEBID depend on a delicate interplay of electron-induced and thermal precursor chemistry with the reactivity of the surface where these reactions take place, and with precursor transport to and on the surface. Novel precursors must be designed specifically for electron beam driven processing and the FEBID process must also be optimized by exploring other types of chemistries. For instance, the chemistry of added purification reagents or catalytic reactions of the underlying surface can be exploited. Such developments must rely on a detailed understanding of all relevant aspects of FEBID. This includes the fundamental electron–precursor interactions leading to precursor fragmentation, surface reactions initiated by these interactions, the design and synthesis of novel FEBID precursors, as well as parameters inherent in the FEBID process. All these factors govern the deposit purity, spatial resolution, and processing speed. The European COST Action CELINA (Chemistry for ELectron-Induced NAnofabrication [9]) has, from 2013 to 2017, stimulated multidisciplinary and multinational collaborative research that aims at understanding the fundamental chemistry of FEBID and improving, on this basis, the performance of FEBID processes. This Thematic Series is a collection of articles that relate to work performed in the framework of CELINA, a majority of which resulted from joint research of groups from at least two European countries.

Among the different subjects that have been pursued within CELINA, the quest for novel precursors is of central importance. Of course, hypotheses about structural elements of better precursors can be developed based on a fundamental understanding of the electron-induced fragmentation of particular precursors [8,10]. Yet, the most systematic approach to this quest is to use quantum chemical calculations to systematically analyze how the fundamental precursor properties vary when the ligand architecture is modified. This can point to promising new target structures for future synthetic efforts [11]. Another approach is to identify molecular subunits that can form stable nonreactive and volatile products upon electron–precursor interaction. The carboxylate group is such a subunit [12–16]. It holds the promise that it may easily fragment to yield thermodynamically stable CO_2_ during electron exposure and thus enhance precursor fragmentation. This expectation is met by a novel fluorinated silver carboxylate precursor that yields deposits with so far unprecedented silver content including 3D structures [15–16]. However, given a favorable structure, even large organic ligands may be removed more easily than previously anticipated, as exemplified by the electron-induced dissociation of benzene–Cr(CO)_3_ [17] and by FEBID using the fluorine-free precursor Cu(tbaoac)_2_ [18]. The most elegant approach to precursor design yet is to use a bimetallic molecular structure to predefine, through the precursor stoichiometry, the composition of an alloy to be deposited by FEBID. In this Thematic Series, a multinational collaboration has reported on a comprehensive investigation of such a novel bimetallic precursor H_2_FeRu_3_(CO)_13_. This work includes all steps of precursor development, from the synthesis to the investigation of its electron-induced fragmentation and surface chemistry, all the way to the actual FEBID process [19].

Precursor fragmentation can be initiated by different types of interaction with the impinging electron. Each type of interaction is effective in a characteristic range of electron energies and leads to different dissociative reactions. It is thus tempting to try to exploit this phenomenon to achieve control over chemistry during electron beam processing. For instance, electron impact ionization initiates fragmentation (named dissociative ionization (DI)) at energies above the ionization threshold while dissociative electron attachment (DEA) occurs already at near-thermal electron energies. Experiments reported in this Thematic Series have aimed at unraveling the role of these processes in FEBID [20]. They use cyclic silane precursors in which DEA has been literally switched on or off by the attachment of suitable atomic side groups. A similar strategy also enables tuning of the processing speed in the fabrication of nanoscale carbon membranes. In fact, an increase of the reaction rate can be achieved by introducing halogen substituents into the molecular precursors that enhance DEA processes [21].

The development of such strategies that enable control over electron-induced processes relies on a detailed understanding of the underlying dissociation reactions. These cannot only be initiated by DEA and DI but may also be the consequence of electronic excitation in the precursor as a consequence of its interaction with an impinging electron. Consequently, fundamental studies on chromium hexacarbonyl [22] and tungsten hexacarbonyl [23] included in this Thematic Series contribute to the important task of building a comprehensive database on the electron-induced dissociation of FEBID precursors. However, it is also important to investigate how these processes change in the presence of a surface or of other molecules. Therefore, it is also shown that DEA at near-thermal energies (which has previously received the most attention among electron-induced precursor chemistry) is suppressed upon increasing aggregation of iron pentacarbonyl [24].

Some precursors that do not a priori perform well in FEBID are, on the other hand, well established with respect to their handling in the process. In these cases, improved deposit purity may be achieved by applying different purification protocols. Recent advancements of such processes are reported in this Thematic Series. This includes a laser-assisted electron beam induced deposition (LAEBID) process in which the laser initiates an additional reaction during deposit growth, which may be further enhanced by simultaneous injection of a reactive gas [25]. Also, it is demonstrated that the metal content of a Au deposit can be significantly increased by continued electron irradiation and a final boost of oxygen plasma cleaning [26] and that the purity of deposits from different metals is enhanced by thermal treatment [27]. However, purification processes in FEBID are also studied with regards to their fundamental chemical processes. For instance, as halide ligands appear promising for future development of FEBID precursors, purification protocols that help to remove them are needed. Using deposits prepared from Pt(CO)_2_Cl_2_, the performance of treatment with atomic hydrogen is thus studied using surface science techniques [28]. Another subject covered is the fundamental chemistry of water-assisted purification processes [29], an approach that has successfully been applied to remove carbon from platinum and gold deposits.

Modeling by different types of simulations is also an important approach to gain a deeper understanding and hopefully better control over FEBID processes. The quality of a deposit depends not only on the chemical properties and processes mentioned so far but also on precursor adsorption states and transport phenomena. The previously developed continuum model describes the precursor adsorption equilibrium and surface diffusion as well as depletion by electron-initiated fragmentation and can thus predict the shape of a deposit. In this Thematic Series, an extension of this model is described that includes multilayer precursor coverage to describe FEBID processes at lower temperatures or with less volatile precursors [30]. Using density functional theory (DFT) calculations, new light was also shed on an experimentally well investigated precursor, namely (CH_3_–C_5_H_4_)Pt(CH_3_)_3_. The interaction of the precursor with a SiO_2_ substrate was modeled to reveal the peculiar role of surface hydroxyl groups. In particular, the partially hydroxylated surface as formed during electron exposure of a fully hydroxylated substrate appears to initiate dissociation of the adsorbed (CH_3_–C_5_H_4_)Pt(CH_3_)_3_ precursor, thus highlighting the importance of the actual chemical nature of the substrate [31].

This Thematic Series is completed by publications on interesting applications of FEBID. This concerns the fabrication and characterization of magnetic cobalt nanospheres on cantilever tips for magnetic resonance force microscopy [32] as well as nanostructures fabricated by a combination of FEBID and autocatalytic growth processes and used as templates for the growth of carbon nanotubes [33].

In summary, the publications collected in the Thematic Series at hand document significant progress in the understanding and implementation of electron-driven precursor chemistry within FEBIP. This progress would not have been possible without the funding received from COST. As obvious from the affiliations on the publications included here, this very valuable networking program has in fact brought together numerous groups from different fields to perform interdisciplinary work towards a common goal. This would have been difficult without the opportunity granted by COST to visit partners abroad at short notice and simply to explore new ideas. Finally, the guest editors particularly thank the *Beilstein Journal of Nanotechnology* and its editorial team in the name of all CELINA participants for the opportunity to publish this Thematic Series and the generous support in doing so. Due to Beilstein’s open access policy, this Thematic Series is a wonderful opportunity to present our results to the entire international community.
